# Supplementary material for: Novel DnaJ Protein Facilitates Thermotolerance of Transgenic Tomatoes
Source: Int J Mol Sci. 2019 Jan 16;20(2):367. doi: 10.3390/ijms20020367 (PMC6359579; doi:10.3390/ijms20020367)
Supplement: Supplementary file 1 [file ijms-20-00367-s001.pdf]

Supplementary Materials

Figure S1. Multiple sequence alignment analysis of SlDnaJ20. AtDjA3 (At3g44110) and PeJ3 (XM_002316443.2) are known as type I J-proteins. SlDnaJ20 lacks a glycine- and phenylalanine-rich region (G/F) domain, zinc finger, and C-terminal domain. The protein sequence of PeJ3 and AtDjA3 contains a J-domain (gold box), a proximal G/F domain (blue box), and a distal zinc finger (CxxCxGxG) domain (red box), followed by less conserved C-terminal sequences (green box).

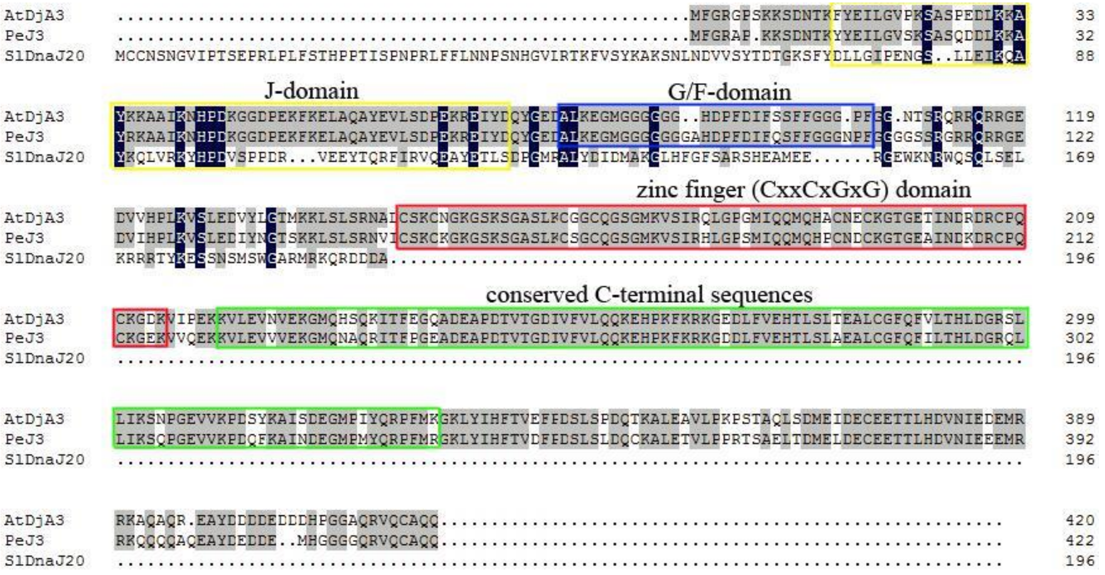

Table S1. Prediction of subcellular localization of SlDnaJ20 using software program TargetP 1.1 (http://www.cbs.dtu.dk/services /TargetP/). Sequence is the amino-acid sequence of SlDnaJ20. Length is the length of the submitted sequence. Numbers given under the cTP, mTP, SP, and other categories represent the probabilities of protein localization in different subcellular regions (cTP, chloroplast transit peptide; mTP, mitochondrial targeting peptide; SP, secretory pathway signal peptide).

Sequence	Length	cTP	mTP	SP	other
SlDnaJ20	196	0.808	0.110	0.048	0.184

Table S2. Prediction of subcellular localization of SlDnaJ20 using software program ChloroP 1.1 (http://www.cbs.dtu.dk/services /ChloroP/). Sequence is the amino-acid sequence of *SlDnaJ20*. Length is the length of the submitted sequence. Score is the output score for the probability of protein localization in the chloroplast. cTP predicts whether a sequence is a cTP-containing sequence, wherein “Y” signifies that the sequence is predicted to contain a cTP. cTP-length is the predicted length of the presequence. cTP, chloroplast transit peptide.

Sequence	Length	Score	cTP	cTP-length
SlDnaJ20	196	0.531	Y	50

Table S3. The primers used for the qPCR reactions.

Gene name	*Sequence* (5′–3′)	
EF1-1α	Forward,	GGAACTTGAGAAGGAGCCTAAG
	Reverse,	CAACACCAACAGCAACAGTCT
SlDnaJ20	Forward,	AGATCGGGTTGAAGAGTAT
	Reverse,	ATAGCTGAGACTGCCAACGGT
SlCuZnSOD	Forward,	TCTTCACCACAACCAGCACT
	Reverse,	CAGTAAGGGGTTTAGGGGTAGT
SlFeSOD	Forward,	GGGAAGTATCACAGGGCGTATG
	Reverse,	GGCTCTCCTCCTCCGTTGG
SlAPX1	Forward,	TGCTGGTACCTACGATGTGTG
	Reverse,	CTGGTGGCTCTGGCTTGTC
SlAPX2	Forward,	GGCTGGTGTTGTTGCTGTTG
	Reverse,	TCAGGCAAGCGACCTTCAAC
SltAPX	Forward,	GAATTCATGACTTCCCTCACAGGC
	Reverse,	AATGCTGATATAAAGCGCAC
HsfA1	Forward,	AGACAACAGCAGCAATCCACT
	Reverse,	ATGTCCTGCTTAATCCTTCGT
HsfA2	Forward,	AATGTTGGTCAGAGTATGAAT
	Reverse,	ATGGCAATGATCTGATTCCT
HsfB1	Forward,	ACTGACGATGTGATATCTT
	Reverse,	AGTTCTCATTGGCGAATTCCCAT
